# Individual variability in neural representations of mind-wandering

**DOI:** 10.1162/netn_a_00387

**Published:** 2024-10-01

**Authors:** Aaron Kucyi, Nathan Anderson, Tiara Bounyarith, David Braun, Lotus Shareef-Trudeau, Isaac Treves, Rodrigo M. Braga, Po-Jang Hsieh, Shao-Min Hung

**Affiliations:** Department of Psychological and Brain Sciences, Drexel University, Philadelphia, PA, USA; Department of Neurology, Northwestern University, Chicago, IL, USA; Department of Brain and Cognitive Sciences and McGovern Institute for Brain Research, Massachusetts Institute of Technology, Cambridge, MA, USA; Department of Psychology, National Taiwan University, Taipei, Taiwan; Waseda Institute for Advanced Study, Waseda University, Tokyo, Japan

**Keywords:** Spontaneous thought, Experience-sampling, Resting-state fMRI, Precision functional mapping

## Abstract

Mind-wandering is a frequent, daily mental activity, experienced in unique ways in each person. Yet neuroimaging evidence relating mind-wandering to brain activity, for example in the default mode network (DMN), has relied on population- rather than individual-based inferences owing to limited within-person sampling. Here, three densely sampled individuals each reported hundreds of mind-wandering episodes while undergoing multi-session functional magnetic resonance imaging. We found reliable associations between mind-wandering and DMN activation when estimating brain networks within individuals using precision functional mapping. However, the timing of spontaneous DMN activity relative to subjective reports, and the networks beyond DMN that were activated and deactivated during mind-wandering, were distinct across individuals. Connectome-based predictive modeling further revealed idiosyncratic, whole-brain functional connectivity patterns that consistently predicted mind-wandering within individuals but did not fully generalize across individuals. Predictive models of mind-wandering and attention that were derived from larger-scale neuroimaging datasets largely failed when applied to densely sampled individuals, further highlighting the need for personalized models. Our work offers novel evidence for both conserved and variable neural representations of self-reported mind-wandering in different individuals. The previously unrecognized interindividual variations reported here underscore the broader scientific value and potential clinical utility of idiographic approaches to brain-experience associations.

## Author Summary

While everyone experiences that their mind “wanders” throughout daily life, the content and form of inner experience is different in different people. In this study, we found that brain activity representing mind-wandering is different in each person, reflecting unique mental experiences. While people consistently engaged the brain’s default mode network (DMN) during mind-wandering, there were inconsistencies in the way that the DMN was engaged and in the other networks throughout the brain that were engaged. Our study highlights that personalized approaches, which require that individuals are sampled more densely than is common in current practice, enable accurate insights into relationships between brain activity and inner experience.

## INTRODUCTION

The brain’s patterns of spontaneous activity are dynamic, yet highly organized, even during task-free periods when external inputs are unchanging (Raichle, 2015). Mind-wandering, often defined as unconstrained, self-generated thoughts that are independent of stimuli and tasks (Smallwood & Schooler, 2015), is a prevalent form of mental activity during these task-free periods and in daily life in general (Kane et al., 2007; Killingsworth & Gilbert, 2010). What people naturally think about, and how thoughts dynamically unfold, are fundamental to cognitive function and mental health (Kucyi et al., 2023). Recent studies have begun to delineate how mind-wandering arises from spontaneous brain activity and have emphasized a key role of the default mode network (DMN) (Christoff et al., 2016; Kam et al., 2022; Kucyi et al., 2023; Smallwood et al., 2021).

The most commonly employed method to study mind-wandering is experience-sampling, wherein people respond to “thought probes” about their subjective level of focus on a cognitive task (Martinon et al., 2019), thereby defining mind-wandering as off-task thought (Christoff et al., 2016). Experience-sampling has been combined online with neuroimaging, revealing an association between mind-wandering with DMN activation (Christoff et al., 2009; Kucyi et al., 2013, 2016; Mittner et al., 2014; Stawarczyk et al., 2011) and broader patterns of whole-brain functional connectivity (Groot et al., 2021; Kucyi et al., 2021; Mittner et al., 2014). These neuroimaging experiments have typically included around 30–40 total thought probes in a single 1- to 2-hr session (Christoff et al., 2009; Groot et al., 2021; Kragel et al., 2016; Kucyi et al., 2013, 2016; Mittner et al., 2014; Sormaz et al., 2018; Stawarczyk et al., 2011; Turnbull et al., 2019; Tusche et al., 2014; Vanhaudenhuyse et al., 2011). Such limited within-subject sampling has led studies to almost exclusively rely on population-level inferences, which harbor the implicit assumption that mappings between brain activity and self-reported mind-wandering are conserved across individuals.

Multiple theoretical and empirical considerations challenge this assumption. First, the content and form of mind-wandering varies substantially across individuals (Heavey & Hurlburt, 2008; Wang et al., 2018), and different subtypes of ongoing thought have distinct neural bases (Sormaz et al., 2018; Van Calster et al., 2017). The specific experiences that tend to occur when different people report off-task thought likely contribute to variable brain-experience mappings. Second, focal lesions within DMN-related regions (Bertossi & Ciaramelli, 2016; McCormick et al., 2018) primarily impact the content of thought without abolishing the ability to engage in mind-wandering (Kam et al., 2022). This points to the possibility of degenerate neural mechanisms (Bernard, 2023), or multiple sets of activity patterns that can give rise to similar self-report outcomes. Third, recent neuroimaging evidence highlights individual variability in neural representations of subjective experiences (Kohoutová et al., 2022), especially when experiences are self-related (Lux et al., 2022). Taken together, these considerations motivate an idiographic (personalized) approach that prioritizes rich, within-person sampling to enable individual-level inferences and tests of group-to-individual generalizability (Fisher et al., 2018).

Importantly, even if there are conserved neural patterns linked to mind-wandering across individuals, neuroimaging studies have not been optimally conducted to detect such patterns. Following common practices in the field, studies have relied on intersubject registration of individual brain images to common-space templates, a method that inevitably results in cross-subject misalignment (Steinmetz & Seitz, 1991). Recent studies using “precision functional mapping” (Gordon et al., 2017; Laumann et al., 2015) have established that the detailed functional anatomy of the DMN and other networks conforms to the unique cortical folding patterns of different brains (Braga & Buckner, 2017; DiNicola et al., 2020; Gordon et al., 2020). Estimating networks within individuals, which can be achieved reliably with dense sampling (Laumann et al., 2015), may unravel why associations between DMN activation and mind-wandering have not always been replicated (Groot et al., 2021) or found consistently within all individuals (Kucyi et al., 2016).

Here we sought to determine whether and how neural activity patterns linked to mind-wandering may be distinct across individuals. We leveraged data from individuals who underwent multiple sessions of functional magnetic resonance imaging (fMRI) where experience-sampling was embedded into a “resting-state” (simple visual fixation) condition, resulting in over 300 thought probes per subject. This paradigm allowed us to examine, with rich individual-level detail, the relationship between natural fluctuations in inner experience and spontaneous brain activity. We applied precision functional mapping and hypothesized that mind-wandering reports would relate (a) reliably to activation of the DMN, including temporal alignment to subjective reports; and (b) unreliably to activation of networks implicated in specific thought features that tend to vary across individuals. We further applied idiographic predictive modeling based on functional connectivity to explore individual-level specificity of whole-brain representations of mind-wandering. We formally compared predictive neural patterns between subjects and tested group-to-individual generalizability using recently developed population-level neuroimaging models of constructs related to mind-wandering (Kucyi et al., 2021; Rosenberg et al., 2020).

## RESULTS

### Mind-Wandering Fluctuations in Multi-Session fMRI

**Figure 1.**
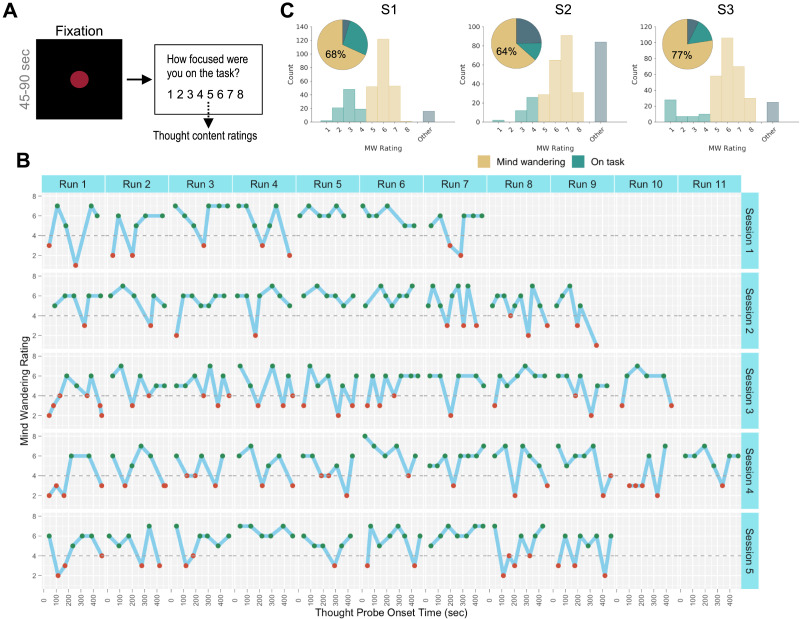
Experience-sampling paradigm and mind-wandering ratings. (A) In fMRI runs involving experience-sampling, subjects were instructed to stare at a red fixation circle. They were intermittently probed every 45–90 s with the question (Q1), “How focused were you on the task?” accompanied by an 8-point Likert scale. A focus rating of 5 or greater was deemed a “mind-wandering trial” and triggered an additional set of questions asking about thought contents. During data analyses, ratings were reverse-coded so that a higher rating indicated more mind-wandering. (B) Example time series of single-subject mind-wandering ratings. The plots show subject S1’s trial-by-trial responses to Q1 across runs within each MRI session. Green and red markers, respectively, indicate trials that were labeled as mind-wandering and non-mind-wandering. Trials in which a different response than 1–8 was submitted (see Methods section) are not shown. (C) Histograms displaying the distribution of responses to Q1, including trials in which a 1–8 rating was provided and “Other” trials in which subjects reported being neither focused on task nor engaged in mind-wandering. Pie charts show the proportions of mind-wandering trials (rating > 4), on-task trials (rating < 5), and “Other” trials in each subject.

Three subjects (S1–3) each completed six fMRI sessions across different days. In one session, they performed functional localizer task runs that we used for precision functional mapping. In each of the other five sessions, they performed 7–11 runs (∼8 min/run) of an experience-sampling task that we used for main analyses. The experience-sampling condition involved a simple visual fixation task, coupled with intermittent thought probes appearing every 45–90 s (Figure 1A). During thought probes, subjects indicated the degree to which they were focused on the fixation task on a 1–8 Likert scale (mind-wandering rating). Alternatively, subjects could respond that they were distracted but were not mind-wandering (e.g., attending to an external stimulus); for the purposes of the present study, we discarded these distraction trials. If subjects reported that they were not focused on the task (rating of 5 or higher on a scale of 1–8), then the trial was deemed “mind-wandering,” and they responded to a series of additional items about thought content and form (see Methods section).

Each subject reported a wide range of task-focus ratings within and across runs and sessions (example shown in Figure 1B). After discarding distraction trials (Figure 1C), 256 or more trials per subject were retained (S1: *n* = 318; S2: *n* = 256; S3: *n* = 316), and over 60% of trials were consistently considered “mind-wandering” based on rating binarization (S1: 68%; S2: 64%; S3: 77%). Depending on the subject, mind-wandering ratings were not correlated, or were weakly correlated, with head motion, time within runs, and trial order within sessions (Table S1A in the Supporting Information). This suggests that there were no consistent associations across subjects between mind-wandering reports and nonspecific factors related to drift over time, such as arousal. However, we present relevant control analyses within subjects where appropriate below.

We further characterized thought content and form for mind-wandering trials based on additional thought probe items that participants responded to for those trials. The additional items inquired about attentional awareness, vividness, perspective (e.g., first- vs. third-person), thought type (recalling, planning, etc.), and content (visual, auditory, emotional, smell, etc.). As expected, specific thought content and form varied across subjects. Predominant thought categories included visual and auditory imagery. Across trials, subjects reported a wide variety of specific mental activities (e.g., recalling, planning, and imagining), perspectives (e.g., first- vs. third-person), and emotions (see Figure S1A in the Supporting Information for a detailed breakdown of thought subcategories). Intersubject comparison revealed that the dissimilarity between each pair of subjects was dependent on thought subcategory; for example, while S1 and S2 were similar in terms of content modality (Supporting Information Figure S1A), they were dissimilar in terms of type of mental activity (e.g., planning vs. recalling) (Supporting Information Figure S1B). Taken together, while mind-wandering was consistently reported frequently across fMRI sessions, specific content and form of thought varied across subjects.

### Activation of the Personally Estimated DMN Is Associated With Mind-Wandering

**Figure 2.**
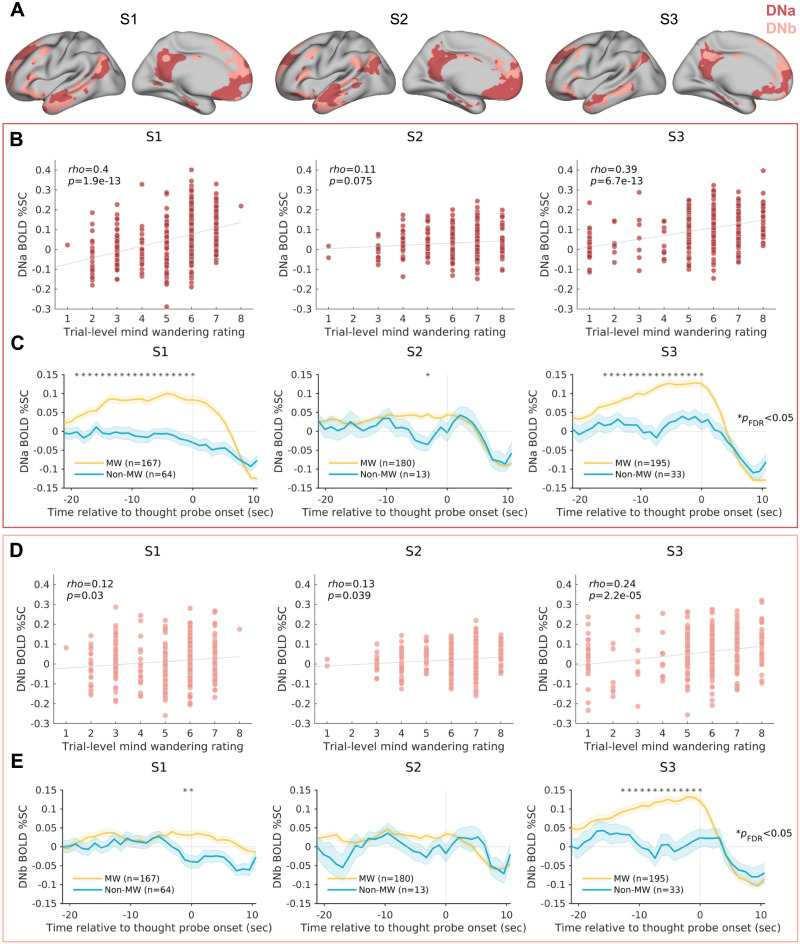
Activation of the DMN, estimated within individuals, is associated with mind-wandering. (A) Two individually estimated networks of the DMN (DNa and DNb) identified among 17 total cortical networks based on multi-session hierarchical Bayesian modeling applied to functional localizer fMRI data. (B) Correlations between trial-by-trial mind-wandering ratings and median DNa blood oxygenation level–dependent (BOLD) percent signal change (%SC) within 10-s pre–thought probe periods. (C) Mean time series plots showing median BOLD %SC in DNa for mind-wandering trials (rating of 6 or higher) and non-mind-wandering trials (rating of 3 or lower) before and after thought probes. Asterisks indicate time points where there was a significant difference in BOLD %SC between trial types (Wilcoxon rank sum test; *p* < 0.05, false discovery rate–corrected for number of samples within 20-s window prior to thought probes). Shaded error bars indicate standard error of the mean. (D) Same as panel (B) but for DNb. (E) Same as panel (C) but for DNb.

We next conducted within-individual analyses of the relationship between trial-by-trial variations in mind-wandering ratings and blood oxygen level–dependent (BOLD) signal activation within the DMN. To precisely map the DMN within individuals, we used multi-session hierarchical Bayesian modeling (MS-HBM), a method for reliable parcellation of the cerebral cortex into a discrete set of labeled systems (Kong et al., 2019). We applied MS-HBM based on functional connectivity analysis of fMRI functional localizer data that was independent from the experience-sampling data (see Methods section). Regardless of how the parcellation level was specified (Supporting Information Figure S2), two DMN-related networks were consistently identified within each subject. These networks demonstrated interindividual variability in spatial arrangements but had organizational motifs that were consistent with previously described fractionation of the DMN into default network A (DNa) and B (DNb) (Braga & Buckner, 2017) (Figure 2A). The two networks neighbored one another and included prominent subregions within medial prefrontal, posteromedial, inferior parietal, and lateral temporal cortices, while a distinguishing feature of DNa was that it was coupled to regions in parahippocampal cortex (Braga et al., 2019).

We extracted the median BOLD percent signal change (%SC) from DNa and DNb within the 10-s periods prior to each thought probe. Within each of the three subjects, we found positive correlations between mind-wandering rating and pre-probe BOLD %SC in DNa, and these correlations were significant within two subjects (S1: Spearman’s *ρ* = 0.40, *p* = 1.9 × 10^−13^; S2: Spearman’s *ρ* = 0.11, *p* = 0.075; S3: Spearman’s *ρ* = 0.39, *p* = 6.7 × 10^−13^) (Figure 2B; see Supporting Information Table S1B for control analyses). For DNb, significant positive correlations with mind-wandering were found within all three subjects (S1: Spearman’s *ρ* = 0.12; *p* = 0.03; S2: Spearman’s *ρ* = 0.13; *p* = 0.039; S3: Spearman’s *ρ* = 0.24; *p* = 2.2 × 10^−5^) (Figure 2D; see Supporting Information Table S1C for control analyses), even though these correlations were weaker than the DNa correlations in two subjects (S1 and S3). For those two subjects, but not for S2, correlations between DNa and mind-wandering were significant over and above the contributions of DNb, as revealed by partial correlations between DNa BOLD %SC and mind-wandering while controlling for DNb BOLD %SC (S1: *ρ*_*partial*_ = 0.44, *p* = 3.3 × 10^−16^; S2: *ρ*_*partial*_ = 0.039, *p* = 0.54; S3: *ρ*_*partial*_ = 0.25, *p* = 6.0 × 10^−6^).

We repeated the correlation analyses using standard-space DMN subnetworks from a commonly used population-derived atlas (Yeo-Krienen 17-network parcellation; Yeo et al., 2011). A standard-space subnetwork commonly referred to as the medial temporal lobe subsystem (Andrews-Hanna, Reidler, Sepulcre, et al., 2010), which overlapped with DNa, showed positive correlations with mind-wandering in all subjects (Supporting Information Figure S3). However, the medial temporal lobe subsystem correlations were weaker than the within-subject DNa correlations, highlighting how precision functional mapping enhanced the ability to detect brain-experience relationships.

Importantly, the analyses presented so far focused on BOLD activation within 10-s pre-probe periods, which is in accordance with prior work (Christoff et al., 2009; Kucyi et al., 2016; Stawarczyk et al., 2011). However, the timing of spontaneous neural activations can vary relative to subjective reports of recalled experiences at the time of a thought probe (Kucyi, 2018). We therefore performed complementary analyses to explore the temporal dynamics of DNa and DNb activations relative to thought probe onsets. We computed BOLD %SC within each subnetwork for trials categorized as “pure” mind-wandering (rating between 6 and 8) or non-mind-wandering (rating between 1 and 3), removing trials with intermediate ratings that may be selected when participants are less certain about their mental experience. This analysis revealed that within DNa, all three subjects displayed specific pre-probe timings where BOLD %SC was significantly greater for mind-wandering than non-mind-wandering trials (all *p*_*FDR*_ < 0.05; Wilcoxon rank sum test) (Figure 2C). In S1 and S3, mind-wandering-related DNa activation was found as early as 18–19 s pre-probe and was sustained until thought probe onset. In S2, there was significant activation only at a time point ∼3 s pre-probe. In all three subjects, DMN deactivation peaked ∼4–6 s after thought probe onset, a characteristic latency that was expected based on the typical timing of neurophysiological responses in the DMN (Raccah et al., 2018) and the hemodynamic delay of BOLD activity (Weissman et al., 2006). Within DNb, only S1 and S3 (but not S2) exhibited pre-probe timings where BOLD %SC was significantly greater for mind-wandering than non-mind-wandering trials (both *p*_*FDR*_ < 0.05) (Figure 2C).

In summary, these findings show that (a) within the 10-s pre-probe window, all subjects demonstrated positive relationships between DMN activation and mind-wandering, with two out of three subjects showing a preferential relationship with DNa relative to DNb; and (b) spontaneous DNa activation was consistently related to mind-wandering within all subjects when accounting for variable timing of activation relative to subjective reports.

### Individual Variability in Functional Networks Activated and Deactivated During Mind-Wandering

We have so far focused on region-of-interest analyses of DMN subnetworks, but mind-wandering has often been associated with activation and deactivation of other networks (Christoff et al., 2016). We therefore next performed within-subject, whole-brain general linear model (GLM) analyses to search for additional brain regions associated with trial-by-trial mind-wandering ratings (z-score normalized within each subject).

**Figure 3.**
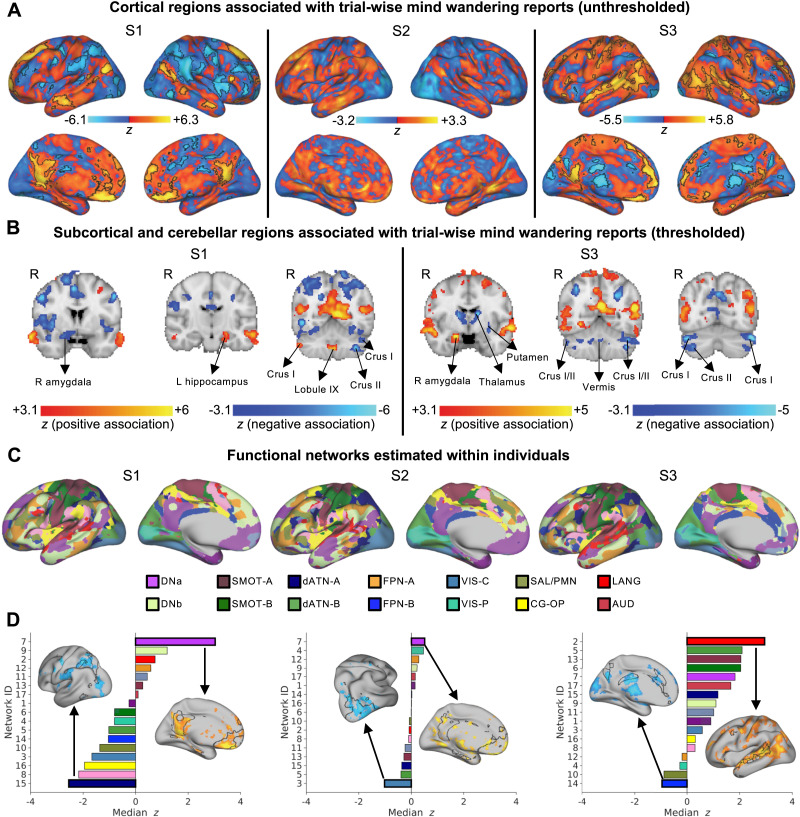
Individual variability in networks activated and deactivated during mind-wandering. (A) Cortical surface plots of unthresholded statistical parametric maps showing regions positively (red–yellow) and negatively (blue–light blue) associated with mind-wandering during 10-s pre–thought probe windows. Maps are based on a whole-brain, within-subject general linear model (GLM) analyses combining all fMRI experience-sampling runs and sessions. Whole-brain corrected, statistically significant clusters are outlined in black (FWE-corrected cluster-determining threshold: Z > 3.1, cluster-based *p* < 0.05). (B) Representative coronal volumes in two subjects who showed significant clusters in subcortical and cerebellar regions. Statistical maps are thresholded to show only significant regions (FWE-corrected cluster-determining threshold: Z > 3.1, cluster-based *p* < 0.05). (C) 17 personalized cortical networks identified with multi-session hierarchical Bayesian modeling applied to functional localizer fMRI data. Labels are provided for 14 of these networks based on consistency with Du et al. (2024). (D) Rank-ordered median z-score (obtained from GLMs) within each one of the networks shown in panel (B). Cortical surface plots are shown for the networks most positively and negatively associated with mind-wandering, respectively; statistical maps are individually thresholded to retain top voxels showing associations, and network outlines are shown in black. AUD = auditory; CG-OP = cingulo-opercular; dATN = dorsal attention network; DN = default network; FPN = frontoparietal network; LANG = language; SAL/PMN = salience/parietal memory network; SMOT = somatomotor; VIS-C = visual central; VIS-P = visual peripheral.

Unthresholded whole-brain statistical maps showing positive and negative associations with mind-wandering are shown in Figure 3A, with suprathreshold significant regions outlined in black (family-wise error rate-corrected cluster-determining threshold: *Z* > 3.1; cluster-based *p* < 0.05). This conservative analysis (i.e., owing to multiple comparisons correction across whole-brain voxels) revealed both significant positive and negative associations with mind-wandering in S1 and S3 but not in S2. In both S1 and S3, significant cortical regions were both within and outside of the DMN. In addition, there were significant associations between mind-wandering and activation within multiple subcortical and cerebellar regions (Figure 3B). For example, in S1, left hippocampus and right amygdala, respectively, were positively and negatively associated with mind-wandering. In S3, right amygdala and bilateral thalamus, respectively, were positively and negatively associated with mind-wandering.

To further characterize cortical areas with respect to networks estimated within individuals, we computed the median z-statistic scores obtained from GLMs within 17 networks independently mapped with MS-HBM (Figure 3C). We rank-ordered these networks based on strength of association with mind-wandering (Figure 3D; see Supporting Information Figure S4 for depiction of group consensus results). For S1, the top (most positively associated) network was DNa, and the bottom (most negatively associated) network was consistent with the dorsal attention network A (dATN-A; including superior parietal lobule and frontal eye fields), a network that classically shows activity that is negatively correlated with the DMN (Fox et al., 2005). For S2, the top and bottom networks, respectively, were DNa and a network involving lateral visual regions. For S3, the top and bottom networks, respectively, were a network that included language-related regions (e.g., left lateral temporal cortex) and a network that was consistent with frontoparietal network B (FPN-B) (Du et al., 2024); the positive associations between mind-wandering and the language network were even stronger than associations with DNa or DNb.

In summary, extending analyses to other networks, there was substantial individual variability in brain activations and deactivations during mind-wandering. To further explore individual variability in broader neural representations at the level of functional network coupling, we next performed a data-driven, idiographic predictive modeling analysis.

### Idiographic Connectome-Based Predictive Modeling of Mind-Wandering

**Figure 4.**
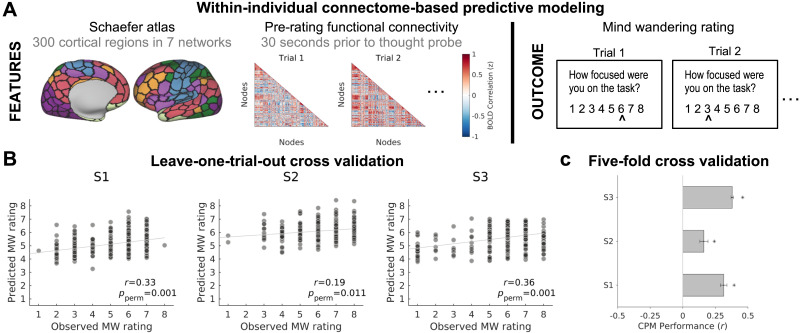
Within-individual connectome-based predictive modeling (CPM) of mind-wandering. (A) Features (left) for CPM included functional connectivity (correlated activity) between regions in a 300-region cortical atlas within 30-s windows prior to thought probes. The CPM outcome to be predicted (right) was mind-wandering rating. (B) Correlation between predicted and observed mind-wandering within each subject based on CPM with leave-one-trial-out cross-validation. (C) Mean correlation between predicted and observed mind-wandering, based on CPM with five-fold cross-validation (see also Supporting Information Table S1D). Error bars show standard deviation of correlations across 120 iterations of cross-validation.

**Figure 5.**
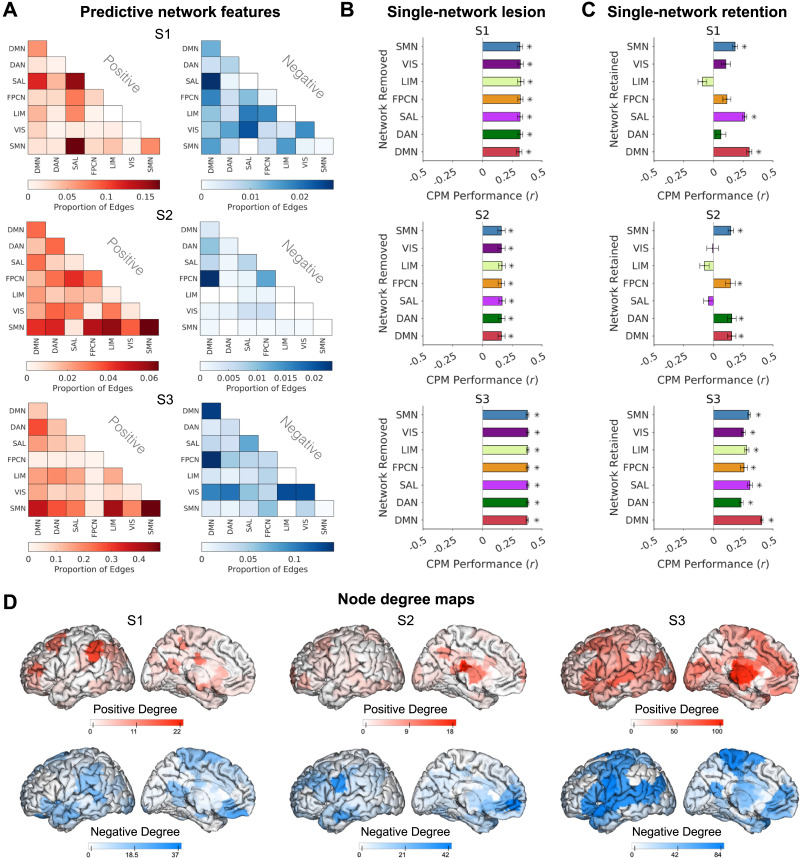
Individual variability in functional anatomy of features important for connectome-based predictive modeling of mind-wandering. (A) Summaries of predictive network features that were selected in CPM to model and predict mind-wandering within each subject. For both positive and negative predictive region-pairs (edges), the proportion of total edges for each pair of networks in the Yeo-Krienen seven-network atlas is shown. (B) Mean correlation between predicted and observed mind-wandering, based on CPMs with different networks virtually lesioned (i.e., all edges involving the “lesioned” network removed for CPM training and testing). (C) Mean correlation between predicted and observed mind-wandering, based on CPMs with all networks removed except for one (i.e., only edges involving the labeled network retained for CPM training and testing). Results shown in panels (B) and (C) are based on five-fold cross-validation. (D) Left hemisphere cortical maps showing node degree (i.e., number of total edge contributions) for regions that positively (red) and negatively (blue) contributed to CPMs within each subject. All error bars indicate standard deviation of correlations across 120 iterations of five-fold cross-validation. DAN = dorsal attention network; DMN = default mode network; FPCN = frontoparietal control network; LIM = limbic network; SAL = salience network; SMN = sensorimotor network; VIS = visual network.

**Figure 6.**
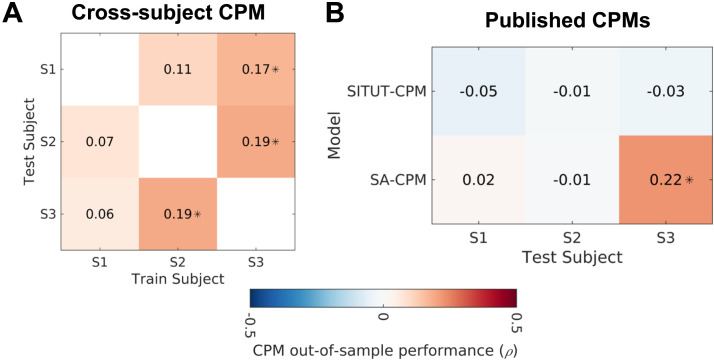
Predicting trial-wise mind-wandering from cross-subject and population-derived connectome-based predictive models. (A) Spearman rank correlation coefficients for predicted versus observed mind-wandering using training data from subjects shown on x-axis and test data from subjects shown on y-axis. (B) Spearman rank correlation coefficients for predicted versus observed mind-wandering using two previously published, population-derived CPMs applied to S1, S2, and S3 as test subjects. Asterisks indicate *p* < 0.05. SITUT-CPM = stimulus-independent, task-unrelated thought CPM; SA-CPM = sustained attention CPM.

Prior work showed that dynamic BOLD functional connectivity can contribute variance to prediction of mind-wandering over and above BOLD activation (Groot et al., 2021; Kucyi et al., 2013; Mittner et al., 2014). We recently applied connectome-based predictive modeling (CPM) to develop a whole-brain, multivariate, functional connectivity model of mind-wandering (defined as stimulus-independent and task-unrelated thought) where model training was performed using combined trials across subjects (Kucyi et al., 2021). Here, in our densely sampled subjects, we performed functional connectivity analyses to address three major questions: (a) Can we generate successful predictions of mind-wandering from CPM performed fully within single subjects (i.e., idiographic CPM) (Figure 4)? (b) If so, are predictive features similar or different across subjects (Figure 5)? (c) Can mind-wandering be predicted within densely sampled subjects using models generated from other densely sampled subjects or from populations of subjects (Figure 6)?

We performed idiographic CPM following procedures that closely matched our prior work (Kucyi et al., 2021). Briefly, we extracted functional connectivity (activity correlation) between all region-pairs in a 300-region cortical atlas within 30-s windows prior to thought probes (Figure 4A). Within training data (a subset of trials), features were selected based on strength of positive or negative association with mind-wandering rating. Within independent, held-out (testing) trials, we compared predicted versus observed mind-wandering and used permutation tests to assess significance (see Methods section).

Idiographic CPMs yielded significant prediction of mind-wandering within each subject when using cross-validation schemes of either leave-one-trial-out (S1: *r* = 0.33, *p*_*perm*_ = 0.001; S2: *r* = 0.19, *p*_*perm*_ = 0.011 ; S3: *r* = 0.36, *p*_*perm*_ = 0.001) (Figure 4B) or five-fold (S1: *r*_*mean*_ = 0.31, *p*_*perm*_ = 0.001; S2: *r*_*mean*_ = 0.16, *p*_*perm*_ = 0.025; S3: *r*_*mean*_ = 0.38, *p*_*perm*_ = 0.001) (Figure 4C; see Supporting Information Table S1D for control analyses). The results were consistent when using the alternative Shen 268-region whole-brain atlas (Shen et al., 2013) (Supporting Information Table S2A) and when using brain basis set modeling (Sripada et al., 2019) instead of CPM (Supporting Information Table S2B). These findings confirm that whole-brain, multivariate patterns of dynamic functional connectivity were predictive of within-subject mind-wandering, even notably in S2 who had not shown significant whole-brain-level activation effects in the univariate GLM analysis (Figure 3A). Having confirmed the success of idiographic CPM, we next compared the features that drove predictions across subjects.

### Idiosyncratic Network Features Predict Mind-Wandering in Different Individuals

For each subject, we obtained CPM-derived positive and negative networks that respectively reflected region-pairs (edges) with functional connectivity strength that was positively and negatively correlated with mind-wandering. In each subject, a large number of positive network edges (S1: *n* = 940; S2: *n* = 322; S3: *n* = 4,954) and negative network edges (S1: *n* = 3,568; S2: *n* = 2,650; S3: *n* = 14,748) contributed to CPMs. To characterize these predictive features, we labeled each node in a region-pair that positively or negatively contributed to a CPM as belonging to one of seven intrinsic networks in the population-based Yeo-Krienen (Yeo et al., 2011) cortical atlas. Each subject showed an idiosyncratic, complex pattern of predictive network features composed of within- and between-network edges involving all seven networks (Figure 5A). For example, while DMN within-network and inter-network edges were included in both positive and negative features in all subjects, the specific edges involved were variable across subjects.

To further determine the contributions of each network to CPM, we performed computational lesion analyses, where all edges from a selected network were removed during feature selection (i.e., leave one network out). When single networks were lesioned, CPM performance always remained significant, regardless of network removed (all *p*_*perm*_ < 0.05; Figure 5B), with minimal effects on performance compared with full models that included all networks. However, the impact of retaining only one network (i.e., leave one network in, or computational lesioning six out of seven networks) was more variable across subjects, with CPM performance remaining significant only for certain single-network models. In S1, S2, and S3, respectively, three (sensorimotor, salience, and DMN), four (sensorimotor, frontoparietal control, dorsal attention, and DMN), and all seven (sensorimotor, visual, limbic, frontoparietal control, salience, dorsal attention, and DMN) single-network models yielded significant performance (all *p*_*perm*_ < 0.05; Figure 5C). This suggests that functional connectivity within some single networks alone is consistently sufficient for successful idiographic CPM performance (sensorimotor network and DMN), while the contributions from other networks are subject-dependent.

The idiosyncratic patterns that were predictive of mind-wandering could be further appreciated via brain maps of high-degree nodes (i.e., regions that participated in many predictive edges; Figure 5D). For example, these maps showed how in S3, high-degree nodes were particularly widely distributed, which explains why single-network retention CPMs consistently yielded significant performance. These high-degree node maps also further highlight how unique the functional anatomy of CPM-derived patterns was in each individual.

### Cross-Subject and Population-Derived Models Exhibit Inconsistent Performance

Our finding of individual differences in predictive network features hint that CPMs generated within individuals (or groups) may not always generalize to other individuals. To further examine this issue, we performed cross-subject CPM: for each pair of subjects, one subject was treated as training data and the other was treated as out-of-sample testing data. This analysis yielded inconsistent performance depending on the subject pair. Half of the train-test pairings resulted in significant cross-subject prediction of trial-wise mind-wandering (all *p* < 0.05, Spearman’s rank correlation; Figure 6A). Cross-subject CPMs between S2 and S3 resulted in bidirectional significant prediction, likely driven by overlapping predictive network features between these subjects such as a negative association between mind-wandering and DMN-frontoparietal control network (FPCN) functional connectivity (Figure 5A). Notably, these two subjects did not consistently report mind-wandering subcategories that were similar to one another, but they did demonstrate similarities in terms of emotional content and perspective-taking (Supporting Information Figure S1B).

We next tested the generalizability of previously published, population-derived CPMs to the densely sampled individuals investigated in the present study. We tested two population-derived CPMs of psychological constructs related to mind-wandering that have previously shown successful prediction of within-subject, trial-wise variability in stimulus-independent, task-unrelated thought (SITUT-CPM) (Kucyi et al., 2021) and sustained attention (SA-CPM) (Rosenberg et al., 2020) (Figure 6B). Applying the SITUT-CPM failed to yield significant out-of-sample predictions of mind-wandering in any of the densely sampled subjects. Applying the SA-CPM revealed significant prediction of mind-wandering in only one of three subjects (*p* < 0.05, Spearman’s correlation). Taken together, cross-subject and population-level functional connectivity models resulted in inconsistent prediction of mind-wandering within densely sampled subjects, highlighting the need for personalized models.

## DISCUSSION

We combined dense-sampling fMRI, experience-sampling, precision functional mapping, and predictive modeling to investigate individual differences in the neural basis of self-reported mind-wandering (defined as off-task thought). We found reliable associations between DMN activation and mind-wandering when estimating networks within individuals and allowing for variable timing of spontaneous BOLD activation relative to subjective reports. Beyond DMN, large-scale networks activated and deactivated during mind-wandering were often distinct across individuals. Idiographic CPM revealed idiosyncratic network coupling patterns that consistently predicted mind-wandering within individuals, while cross-subject and population-derived models performed inconsistently when applied to densely sampled individuals. Collectively, our findings detail previously unrecognized, substantial interindividual variations in neural correlates and predictors of mind-wandering. We discuss the implications of our findings for conceptualizing mind-wandering and the brain, interpreting resting-state brain activity, and advancing clinical applications.

### Neural Basis of Mind-Wandering: From Populations to Individuals

While it is recognized that mind-wandering is supported by multiple brain networks and neuromodulatory systems, the role of the DMN has received the most attention to date (Christoff et al., 2016; Kucyi et al., 2023; Mittner et al., 2016). Some of the first-described functional properties of the DMN were that this network deactivates during externally oriented task performance (Raichle et al., 2001; Shulman et al., 1997) and activates during mental experiences that are common during mind-wandering such as thinking about the past or future with self-related or social content (Andrews-Hanna et al., 2014). Neuroimaging studies (Andrews-Hanna, Reidler, Huang, et al., 2010; Mason et al., 2007), including those with online experience-sampling (Christoff et al., 2009; Kucyi et al., 2013, 2016; Mittner et al., 2014; Stawarczyk et al., 2011), subsequently offered evidence of a relationship between DMN activation and self-reported thoughts that are stimulus-independent and task-unrelated. Human intracranial recordings have further confirmed that neuronal population activity within DMN regions increases during attentional lapses (Kucyi et al., 2020). However, some fMRI studies have failed to show relationships between DMN activation and mind-wandering in group-level analyses (Groot et al., 2021), or in certain (non–densely sampled) individuals (Kucyi et al., 2016), or have alternatively shown relationships with more specific features of thought such as subjective level of detail (Sormaz et al., 2018).

We showed that when boosting within-subject statistical power, as well as accounting for personalized functional anatomy and BOLD temporal dynamics, DMN activation was reliably associated with mind-wandering (defined as off-task thought) during a simple visual fixation task. In two of three subjects (S1 and S3), DNa exhibited stronger associations than DNb. The functional differences between these two subnetworks remains an active research topic. One dense-sampling fMRI study showed that while DNa was recruited during remembering and imagining the future, DNb was alternatively recruited during social thoughts about others’ experiences (theory of mind) (DiNicola et al., 2020). The subcategories of mind-wandering that our participants reported suggest that all individuals engaged in recalling, planning, and imagining (Supporting Information Figure S1A), which may explain consistent DNa recruitment. While thought probes in this study did not inquire directly about theory of mind, we speculate that the more “equal” recruitment of DNa and DNb found in one participant (S2) could suggest a tendency to engage in socially oriented thoughts. Further, targeted investigations may address the distinct roles of DNa and DNb in spontaneous thought.

Our within-subject analyses revealed variability in the time courses of DNa and DNb activation relative to subjective reports, an effect that has not been appreciated in prior studies because of limited within-individual sampling. Interestingly, mind-wandering-related activation in these networks could sometimes be detected in BOLD activity up to ∼20 s prior to thought probe onsets (i.e., ∼25 s when accounting for hemodynamic delay). Relatedly, prior studies (at the group level) have revealed trends of increased DMN activation (Eichele et al., 2008) and EEG alpha power (O’Connell et al., 2009) up to ∼20–30 s prior to task performance errors. One potential interpretation of prolonged pre-probe DMN activation is that random-interval thought probes often capture mind-wandering when a person is already deeply immersed in self-generated experiences. In future studies, mind-wandering episodes may be captured more efficiently via real-time fMRI detection of DMN activation to potentially reduce the amount of within-subject sampling needed to identify brain-experience relationships.

Beyond DMN, we found that functional networks and regions most activated during mind-wandering were variable across subjects. One subject (S1) showed an activity pattern that largely aligns with expectations from population-level neuroimaging: The dorsal attention and salience networks, typically associated with externally oriented attention (Corbetta & Shulman, 2002; Uddin, 2015), were most activated during task-focus, while DMN subnetworks were most activated during mind-wandering. This subject also showed mind-wandering-related hippocampal activation, potentially hinting at support for the hypothesis that hippocampal sharp-wave ripple events trigger DMN activation to initiate spontaneous thought (O’Callaghan et al., 2021). Another subject (S3) showed a whole-brain pattern that was not necessarily anticipated from population-level neuroimaging. The pattern included prominent activations of the language network and thalamic deactivations. As this subject reported a relatively high proportion of thoughts with auditory-language content (Supporting Information Figure S1A), we speculate that thalamic deactivation reflected dampening of sensory input (i.e., perceptual decoupling; Schooler et al., 2011) to maintain attention toward self-generated inner speech. The third subject (S2) had weaker effect sizes than the others in terms of relationships between mind-wandering and activation of both the DMN and other networks. These weaker effects may be due to multiple factors, such as decreased statistical power (fewer analyzable trials available in this subject), “noisier” introspective access to thought processes, or increased within-subject variability of thought subtypes.

We explored the broader role of intra- and inter-network interactions through network-based predictive modeling within individuals. This analysis revealed contributions of various intrinsic networks that were not necessarily captured in the BOLD activation analyses. This divergence is consistent with prior work demonstrating complementary, rather than overlapping, contributions of regional activation and functional connectivity to prediction of mind-wandering (Groot et al., 2021; Mittner et al., 2014). Population-based predictive modeling analyses of mind-wandering have emphasized the importance of features that involve DMN within-network and between-network interactions (Groot et al., 2021; Kucyi et al., 2021; Mittner et al., 2014), though broader interactions beyond DMN contributed substantially to the recently reported SITUT-CPM (Kucyi et al., 2021). Our densely sampled subjects each showed idiosyncratic predictive networks that did not fully align with population-derived models. For example, while DMN-FPCN connectivity was positively related to mind-wandering in the SITUT-CPM (Kucyi et al., 2021), two subjects here showed a largely opposite pattern for DMN-FPCN edges. Though within-DMN features were sufficient for prediction within each subject, computational removal of the DMN had a minimal impact on prediction performance, and there were also other networks that were alone also sufficient for prediction performance. For example, the sensorimotor network, which has been previously linked to individual differences in mind-wandering content (Mckeown et al., 2020), was sufficient for prediction within all subjects.

One implication of our findings is there could be multiple neural “paths” to mind-wandering (i.e., degeneracy). This possibility is in part supported by recent studies that demonstrated that EEG predictors of mind-wandering are distinct across individuals (Dhindsa et al., 2019; Dong et al., 2021). Moreover, a recent application of fMRI multivariate pattern analysis revealed that person-specific, compared with population-derived, neural models could better decode affective valence when people reflected on concepts in spontaneous thought that were high in self-relevance (Lux et al., 2022). As degenerate mechanisms have been described at multiple levels of the nervous system (Prinz et al., 2004; Tononi et al., 1999), there is a need to more deeply investigate their potential importance to brain-experience relationships.

### Implications for Interpreting Spontaneous Brain Activity

Our findings build on research linking spontaneous brain activity to ongoing cognition (Kucyi et al., 2018; Liu et al., 2022). There has remained debate in the field over whether “thinking,” as contrasted with intrinsic physiological processes such as offline plasticity and homeostasis, contributes meaningfully to functional network patterns that are observed in BOLD spontaneous activity (e.g., in “resting-state” fMRI) (Finn, 2021; Gratton et al., 2018; Kucyi, 2018; Laumann & Snyder, 2021; Lurie et al., 2020). On the one hand, resting-state functional connectivity patterns are highly stable within individuals across multiple timescales (Finn et al., 2015; Gratton et al., 2018). On the other hand, functional connectivity fluctuates on the order of seconds (Chang & Glover, 2010; Liu & Duyn, 2013), and rapid fluctuations encode spontaneous behavior (Benisty et al., 2024). Yet in typical resting-state fMRI studies, measures of ongoing cognition or behavior—especially of an individual’s unconstrained experiences—are rarely obtained (Gonzalez-Castillo et al., 2021).

In a few prior works that involved limited individual-level sampling (e.g., single-session fMRI), online experience-sampling during “rest” has revealed group-derived neural correlates of ongoing thought (Van Calster et al., 2017; Vanhaudenhuyse et al., 2011). Our findings advance upon those works, highlighting clearly, at the individual level, that dynamics of spontaneous BOLD activation and functional connectivity are relevant to reportable mental experiences. The effect sizes that we achieved with idiographic predictive modeling were often substantially higher than those typically reported for population-derived functional connectivity models of self-report outcomes (Chen et al., 2022; Kucyi et al., 2021), while we found that cross-subject and population-derived predictive models often failed when applied to individuals. These findings indicate that fluctuations in resting-state BOLD functional connectivity may more closely relate to ongoing experience than has been appreciated, yet such brain-experience relationships may not be detected when relying only on population-derived inferences.

Our findings may even suggest that functional connectivity “fingerprints” (i.e., individual-specific, state-independent patterns; Finn et al., 2015) are driven by tendencies to engage in certain forms of thought, which could be conceptualized as “thought fingerprints.” Support for this idea comes from a recent study demonstrating that when spontaneous thoughts were abolished owing to deep general anesthesia, functional connectivity patterns became less interindividually distinguishable (Luppi et al., 2023). People often report certain patterns of thought that are relatively stable (Ho et al., 2020) (despite influence of context; Mulholland et al., 2023), which may imply that during resting states, thought trajectories tend to “default” toward recurring patterns that an individual is predisposed to experiencing. These considerations promote the idea that resting-state fMRI interpretability may be enhanced through incorporating annotations of experiences (Finn, 2021; Gonzalez-Castillo et al., 2021; Kucyi, 2018).

### Clinical Implications of Personalized Neuroimaging

The limited group-to-individual generalizability illustrated here has implications for clinical efforts that aim to identify neuroimaging-based biomarkers or treatment targets. The way that ongoing thoughts tend to unfold for a given individual carries important consequences for mental health (Kucyi et al., 2023). Recently developed, population-derived neuroimaging predictive models of ongoing thought have been applied to patients diagnosed with psychiatric illness. The SITUT-CPM predicted excessive mind-wandering in adults with attention-deficit/hyperactivity disorder (Kucyi et al., 2021). A resting-state fMRI model of trait rumination predicted depression scores in adults with major depressive disorder (Kim et al., 2023). However, such model predictions do not succeed in all individuals, and population-derived predictive models are most likely to fail in individuals who defy sample stereotypes (e.g., owing to sociodemographic and/or clinical factors; Greene et al., 2022). As such, population-derived approaches are not likely to yield one-size-fits-all biomarkers (Dhamala et al., 2023).

An idiographic approach, as taken here, may offer an alternative way to identify state-related biomarkers of thought processes in a personalized manner. The potential clinical impact of this approach was illustrated in a recent *n*-of-1 study where intracranial electrophysiology was combined with experience-sampling in a patient with treatment-resistant depression. A neural model of within-individual fluctuations in subjective mood was used to guide closed-loop deep brain stimulation and was predictive of the effects of stimulation on mood improvement (Scangos et al., 2021). Idiographic neuroimaging models of spontaneous experience, as developed here, may similarly be used to guide personalized treatments in a noninvasive manner.

### Limitations and Future Directions

While our work illustrates the benefits of a personalized neuroimaging approach to the study of mind-wandering, it also highlights several limitations and opportunities for future research surrounding the issues of interpretability, intraindividual reliability, and—at a practical level—scalability of a personalized approach. Regarding interpretability, while we have speculated about potential reasons that distinct brain regions and networks are associated with mind-wandering in different individuals, our study does not fully shed light on why such differences are found. For example, the variations may reflect differences in cognitive and affective characteristics of spontaneous thought that we did not measure in detail. Future studies should be more optimally designed to capture the specific content and dynamics of such thought characteristics, for example by combining experience-sampling with actively generated personal narratives (Kim et al., 2024) or using think-aloud paradigms (Raffaelli et al., 2021) to derive more granular information about thought qualities. Drawing from larger subject samples, future research may determine whether individuals who experience similar types of thoughts also show similar brain-experience relationships.

Even though our subjects were sampled relatively densely, our study was not optimally designed for examining intrasubject reliability of relationships between mind-wandering and neural activity. The content and form of mind-wandering may fluctuate from day to day, for example owing to factors such as stress or major life events. While it is difficult to fully account for such variations, future studies should examine the stability of within-individual mappings between brain activity and mind-wandering. Such mappings could be unstable over time because of factors such as measurement error or meaningful variation in the relationship between brain activity and behavior/experience (Flournoy et al., 2024). This issue is especially of importance when considering recent fMRI evidence that neural representations of the same sensory stimuli can drift over time (e.g., across sessions over the course of months; Roth & Merriam, 2023).

Finally, there are limitations concerning the scalability of our approach given that extensive data were needed per individual, necessitating higher participation demand than is typical for neuroimaging studies (although participants in this study found the tasks to be feasible and acceptable). The issue of weighing costs versus benefits of dense sampling has been discussed extensively in the precision fMRI literature. It has been argued that cost-benefit analyses may justify intensive sampling in certain clinical contexts (Gratton et al., 2020; Laumann et al., 2023). Within the context of mind-wandering, the optimal amount of data per individual remains unknown, although this amount is likely greater than that needed to simply map personalized functional networks. Approaches such as real-time fMRI for “stealth assessments” of mind-wandering episodes, and innovative data analyses, are strategies that may be explored in the future to enhance within-subject statistical power, reduce participant burden, and improve feasibility across broader contexts.

## METHODS

### Participants and General Procedure

Three study participants (S1, S2, and S3; age range: 25–29, one male) were recruited from the Duke–National University of Singapore (Duke-NUS) Medical School community. Each participant completed six 2-hr scanning sessions on different days (each separated by roughly 1 week). All participants had normal or corrected-to-normal vision and reported no neurological, psychiatric, or sleep disorders. Participants provided informed consent for procedures approved by the National University of Singapore institutional review board.

The full procedures of this study were reported elsewhere (Hung & Hsieh, 2022). Participants each completed six sessions of neuroimaging, which included functional localizer and experience-sampling conditions. Data analyses reported here are independent from those applied to this dataset previously, as prior work focused on a subset of experience-sampling trials that involved specific categories of visual and auditory imagery (Hung & Hsieh, 2022).

### Neuroimaging Data Acquisition

Neuroimaging scans were performed with a 3-Tesla Siemens Prisma scanner equipped with a 12-channel head coil at Duke-NUS Medical School in Singapore. Functional magnetic resonance imaging (fMRI) runs were performed with a gradient echo-planar imaging multiband sequence (repetition time: 1.06 s, echo time: 32 ms, flip angle: 61°, field of view: 1,980 × 1,980 mm, 2 × 2 mm in-plane resolution, 2 mm slice thickness) with collection of 36 slices aligned with the AC-PC plane. Approximately 10 runs were collected per session for each participant. In each session, a T1-weighted anatomical image was also acquired (repetition time: 2.3 s, inversion time: 900 ms, flip angle: 8°, field of view: 256 × 240 mm, 192 slices, 1 × 1 × 1 mm voxels).

### Experience-Sampling Paradigm

Participants completed 46 fMRI runs of a fixation task with experience-sampling across five neuroimaging sessions (7–11 runs per session; ∼5.8 hr total). Before scanning, participants went through task training and instructions. The main task of the experiment was an 8-min fixation task with intermittent experience-sampling. Participants were instructed to stare at a fixation cross without thinking of anything in particular. Any thought unrelated to the task was defined as mind-wandering. Thought probes would appear at random intervals between 45 and 90 s. The first thought probe item inquired about the degree to which attention was focused on the task on a Likert scale of 1–8. Ratings between 1 and 4 indicated that their mind was wandering, while ratings between 5 and 8 indicated task focus. There was also an option to provide a “9” rating to indicate being distracted but not mind-wandering (e.g., attending to external stimuli such as scanner acoustic sounds). These “distracted” trials were removed from analyses. For the purposes of intuitive interpretation, we flipped the rest of the mind-wandering ratings prior to all analyses (i.e., subtracted by 9) such that greater mind-wandering was associated with a higher rating.

If participants reported being focused on the task at the first probe, they were immediately brought back to the fixation task. Conversely, if participants indicated mind-wandering for the first thought probe item, then a series of additional items followed to elaborate on thought content and form. Thought probe items 2–5 probed (2) attentional awareness on scale of 1–8; (3) vividness on scale of 1–8; (4) perspective: first person, third person, or not sure; (4) thought type: recalling, planning, imagining, reasoning/thinking, or distracted; and (5) content description: visual, auditory, emotional, smell, taste, and bodily sensation (check all that apply). If categorization included visual thoughts, participants would next specify whether the imagery included faces, body parts, animals, plants, natural scenes, artificial scenes, motion, nonliving objects, or food. If categorization included auditory thoughts, participants would specify whether the thought included animal sounds, language, music, or other sounds. Lastly, if categorization included emotional thoughts, participants would specify whether they were happy, surprised, angry, disgusted, sad, or afraid. There was no time limit for answering questions.

In addition to the preprogrammed thought probes, participants had the option of prompting probes on their own if they caught themselves mind-wandering before a probe appeared; however, there were no self-caught mind-wandering prompts reported by any of the three subjects. Intervals between probes were informed by the participants’ prior run performance in an attempt to catch one instance of mind-wandering every minute. If participants reported less mind-wandering with the initial interval settings in the previous block, the probe interval length was increased in the next block. Conversely, if participants reported more mind-wandering than anticipated, interval length was decreased.

During the task, eye tracking was used to detect drowsiness. Prolonged eye closure twice in a session was interpreted as excessive drowsiness and the run was ended. The participant was then given a break before beginning the next run. Overall, 350 thought probes per subject appeared. Participants navigated probe response options using a button box in one hand, and they confirmed their selection(s) using another button box in their other hand.

Importantly, before the MRI experiment, each participant underwent a behavioral mock session with the same experience-sampling task, which was intended to familiarize them with the procedure. They were instructed to utilize the 8-point scale fully. In this training, the participants also learned that for the first question (regarding mind-wandering), a rating of 4 versus 5 was the cutoff for mind-wandering versus non-mind-wandering reports since only the mind-wandering reports prompted follow-up questions. It is thus likely that all participants incorporated this cutoff in their reports during the experimental sessions. While it is impossible to completely rule out that participants used the scale differently, this prior training aimed to minimize individual differences in scale use.

### Functional Localizer Paradigms

Across one or two of the six sessions, participants each completed 10 total fMRI runs of functional localizer tasks (51.4 min total). These tasks were run for the original purpose of identifying regions of interest involved in visual and auditory processing (Hung & Hsieh, 2022). In the present study, the functional localizer data were used for the purpose of mapping personalized functional networks.

Retinotopic mapping scans were collected in three runs. In each run, stimuli of flashing checkerboard wedges were presented in different orientations across three conditions. These conditions were pseudo-randomized across six 20-s blocks with 20-s fixation preceding and succeeding each block. Across all fixation conditions, subjects were instructed to use buttons to indicate the color of the central cross as it switched between red and green. In the horizontal condition, two wedges appeared in-line horizontally beneath the fixation cross. In the vertical condition, another two wedges appeared in-line vertically beside the fixation cross. In the diagonal condition, four wedges appeared around the fixation cross along the diagonal axis. Each condition repeated twice per run.

Three additional runs of six 20-s blocks and 20-s fixation were performed for auditory mapping. In each block, successive tones from two of three frequency ranges—high (2,370–5,900 Hz), mid (880–2,170 Hz), and low (340–870 Hz)—were played in 10-s cycles. Within a cycle, the frequency of the tone was increased linearly for 5 s before decreasing for another 5 s. Subjects were instructed to press a button at the tone’s peak frequency.

Four runs of 16 20-s blocks were run to localize the visual regions involved in processing faces, scenes, and objects. Participants were presented with grayscale images of faces, scenes, common objects, and scrambled objects, with four blocks dedicated to each category.

### Behavioral Data Analyses

Individual differences in experience-sampling ratings were summarized by visualizing relative proportions in mind-wandering content and frequency across all runs and sessions. Mind-wandering frequency was summarized by visualizing the relative proportion of mind-wandering trials to on-task trials. Mind-wandering trials were defined as trials where the response to the probe question “How focused were you on the task?” was greater than or equal to 5 (after reverse-coding). Mind-wandering content was summarized by visualizing the relative proportion of responses across subjects to the probe question “Check all that well describes the contents of your mind-wandering” (Supporting Information Figure S1A).

We examined whether mind-wandering ratings (response to first thought probe item) were associated with time on task (within runs and sessions) or head motion within each subject. Within-session time on task was defined as the ordered thought probe number within a session. Within-run time on task was defined as the onset time (in seconds) of a thought probe within a run. Head motion was defined as mean framewise displacement, as computed with the FMRIB software library (FSL) linear image registration tool (Jenkinson et al., 2002), across the 28 repetition times (TRs) prior to each thought probe. We computed Spearman’s rank correlation coefficients between mind-wandering ratings and each of these metrics within subjects (significance set at *p* < 0.05, two-tailed).

To assess the degree to which subjects were similar to one another in their responses to thought probes, we performed a distance calculation on responses to categorical probe questions. The amount of times that a subject responded to each option for a given probe question was counted across all mind-wandering events. This yielded a vector of counts for each subject and for each probe question, where each element in the vector was a count of responses from a subject to a particular option for a probe question (e.g., [142, 79, 8] is the count vector of responses from Subject 1 to probe question “Perspective” tallying the counts to options “First person,” “Third person,” and “Not sure,” respectively). The distance between two subjects for a probe question was compared by taking the Euclidean distance (i.e., square root of the sum of squared differences) between the respective count vectors. Lower distances suggest that two subjects responded more similarly to a given probe question throughout the experiment.

### fMRI Preprocessing

Preprocessing of all 46 experience-sampling fMRI runs within each subject was performed using a combination of tools from FSL v6.0.3 (Jenkinson et al., 2012), the analysis of functional neuroimages (AFNI v21.3.06) package (Cox, 1996), and independent components analysis for automatic removal of motion artifacts (ICA-AROMA). Procedures were similar to those previously described (Kucyi et al., 2016), though an exception was that we took extra steps to optimize within-subject alignment of all fMRI runs across sessions (Braga & Buckner, 2017). We created a common BOLD template by computing a mean volume from all runs and sessions of a single participant. We then aligned each run to the common BOLD template using the MCFLIRT tool in FSL, thereby performing motion correction and moving all runs into the same common space. Using each subject’s T1 structural scan from their first MRI session, we performed segmentation with FSL’s FAST tool to create white matter (WM), cerebrospinal fluid (CSF), and gray matter (GM) templates. Using FSL’s FEAT to process fMRI data, we performed brain extraction (BET), spatial smoothing (5-mm full-width at half-maximum kernel), linear registration (FLIRT) between BOLD and T1 spaces, and nonlinear registration (FNIRT) between BOLD and standard MNI152 space. Motion artifacts were then addressed with ICA-AROMA, which has been shown to improve sensitivity and specificity for both resting-state and task-based fMRI analyses (Pruim et al., 2015). This included running ICA with automatic dimensionality estimation using FSL’s MELODIC tool and regression of automatically detected motion-relevant components of fMRI data. Next, we set a threshold of 198 cm^3^ and 20 cm^3^ for WM and CSF probabilistic maps, deleted below-threshold volumes, and regressed out the mean WM and CSF time series from fMRI data. We applied a high-pass temporal filter (0.01 Hz cutoff) for univariate activation analyses or a bandpass filter (0.01–0.1 Hz) for functional connectivity analyses described below. The preprocessed data were transformed to MNI152 space using linear registration, and percent signal change (%SC) of voxel intensity values was computed by dividing the difference of values by the mean value in each run; the quotient was then multiplied by 100 for the final percent value.

The functional localizer data were preprocessed with a different preprocessing pipeline to enhance consistency with pipelines that were used to validate the MS-HBM approach for personalized functional network mapping (Kong et al., 2019). We used publicly available fMRI preprocessing code shared by the Computational Brain Imaging Group (https://github.com/ThomasYeoLab/CBIG/tree/master/stable_projects/preprocessing/CBIG_fMRI_Preproc2016). Structural data (T1 scan from first session) were reconstructed into surface mesh representations using the *recon-all* command implemented in FreeSurfer v6.0.0. Motion correction was performed using rigid body translation and rotation in FSL. Structural and functional images were aligned using boundary-based registration (Greve & Fischl, 2009). We performed linear detrending and regressed out nine regressors plus their temporal derivatives, including global signal, averaged WM signal, averaged CSF signal, and six motion correction parameters. A bandpass filter of 0.01–0.1 Hz was applied. Preprocessed fMRI data were then projected to the FreeSurfer fsaverage6 surface mesh, and spatial smoothing was performed on the surface with a 6 mm full-width half-maximum kernel.

### Mapping Personalized Functional Networks

We applied the MS-HBM approach (Kong et al., 2019) to each subject’s functional localizer fMRI data to generate personalized functional connectivity networks, using publicly available code (https://github.com/ThomasYeoLab/CBIG/tree/master/stable_projects/brain_parcellation/Kong2019_MSHBM). While our functional localizer data involved multiple conditions, we deemed the combined data to be suitable for personalized network mapping because functional networks, as estimated over tens of minutes or more, show high stability within individuals regardless of task conditions (Gratton et al., 2018; Wang et al., 2015). The MS-HBM method utilizes prior distributions of each cortical vertices’ functional connectivity profiles from various hierarchies (e.g., group level; intersubject level; and intrasubject, inter-session level) to estimate individual-specific brain networks unique to each subject (Kong et al., 2019).

Model priors were computed based on initial binarized functional connectivity profiles calculated for multiple equally spaced cortical vertices, or designated points across the surface of the cortex. These functional connectivity profiles were defined as the top 10% of all correlations between the preprocessed BOLD signal at each cortical vertex and those of a predefined set of 1,175 regions spanning the cerebral cortex. To estimate the first prior hierarchy (a group-level network profile), the average binarized connectivity profile of all cortical vertices of all subjects was computed. This was then used to model the next prior (intersubject network profile) for each subject, which considers the functional connectivity variability between subjects. The following prior (intrasubject, inter-session network profile) compares the average connectivity profiles of all vertices from one session of a subject to those of that subject’s other sessions. Finally, the connectivity variance between different regions of the same network as observed in a single session of a subject (intra-session, inter-region profile) was modeled.

These priors then informed the model to stabilize estimations of individually parcellated whole-brain networks, such that if the connectivity profile of a given vertex is most similar to the connectivity profile of a certain brain network at a specific prior hierarchy, the vertex would then be assigned to that network. For each subject, each cortical vertex was assigned to one of *k* = 17 networks (where *k* is number of clusters), consistent with the 17-network solution of the population-level Yeo atlas (Yeo et al., 2011). This approach of individually parcellating brain networks allowed us to analyze each subject’s brain activity within their own personalized cortical map. To inspect the consistency of networks obtained with MS-HBM in our dataset, we repeated the procedures with the algorithm constrained to *k* values of 13–16.

### Region-of-Interest Analyses of Default Mode Network

We visually inspected the 17 networks obtained with MS-HBM within each subject and identified the personalized networks that were most consistent with the previously described DMN subnetworks known as DNa and DNb (Braga & Buckner, 2017; Braga et al., 2019). We projected the network masks from fsaverage6 surface space to the MNI152 volumetric space of the preprocessed experience-sampling fMRI data. We then extracted the median BOLD %SC time series, separately from DNa and DNb voxels. For each thought probe across all runs and sessions, we computed BOLD %SC within each network averaged across nine TRs prior to thought probes (i.e., corresponding to 9.54 s). We computed Spearman’s rank correlation coefficient between trial-wise mind-wandering ratings and the ∼10-s pre-probe BOLD %SC values for the DNa and DNb (significance set at *p* < 0.05, two-tailed). We then repeated these analyses using alternative, standard-space DMN subnetworks instead of the personalized DNa and DNb subnetworks. For this comparison analysis, we extracted BOLD %SC from three DMN subnetworks in the Yeo17 population-level atlas (Yeo et al., 2011), which have been described as “Core DMN,” “dorsomedial prefrontal cortex subsystem,” and “medial temporal lobe subsystem” (Andrews-Hanna, Reidler, Sepulcre, et al., 2010).

Beyond our analyses that focused on averaged BOLD %SC within ∼10-s windows prior to thought probes, we performed a complementary analysis of TR-by-TR temporal dynamics within DNa and DNb. For each subject, we split trials into “pure” high mind-wandering (rating between 6 and 8) and non-mind-wandering (rating between 1 and 3) while discarding trials that had intermediate ratings. At each TR between −20.14 and 0 s prior to thought probes (i.e., 20 TRs), we computed the average of median BOLD %SC within DNa and DNb across trials for each trial type. At each of these 20 TRs (and for each subnetwork), we performed Wilcoxon rank sum tests to compare BOLD %SC between high and low mind-wandering trials. Significance was set at *p* < 0.05 (two-tailed), false discovery rate corrected for multiple comparisons across 20 TRs. For visualization purposes, we further plotted average time courses of high and low mind-wandering trials prior to and after the ∼20-s window of interest.

### Whole-Brain General Linear Model Analyses

We performed within-subject, voxel-wise general linear model (GLM) analyses in FSL to search the whole brain for regions associated with mind-wandering ratings. In this analysis, we normalized ratings within each subject (across all sessions/trials) by computing z-scores. For each experience-sampling fMRI run, we ran a first-level GLM on preprocessed data using FMRIB’s improved linear model prewhitening. This model included a parametric regressor for within-subject z-scored mind-wandering rating at the 10-s period prior to thought probes (convolved with a gamma hemodynamic response function). Contrasts were performed to identify voxel clusters associated positively or negatively with mind-wandering rating. Owing to lack of variation in mind-wandering ratings within a small number of runs (S1: 0 runs; S2: 1 run; S3: 1 run), those runs were excluded from GLM analyses. The first-level GLM statistical maps were submitted to higher level, within-subject GLMs that combined all runs and sessions and were performed with FMRIB’s local analysis of mixed effects (FLAME) 1 + 2 (cluster-based thresholding at *Z* > 3.1 and family-wise error corrected *p* < 0.05). For visualization purposes, GLM results were projected to the fsaverage6 cortical surface and displayed with Connectome Workbench software (Marcus et al., 2011). To provide further interpretation of whole-brain GLM results in terms of personalized cortical networks, we extracted the median z-statistic score within each network identified by MS-HBM.

### Within-Subject Connectome-Based Predictive Modeling

We performed idiographic (within-subject) CPM (Shen et al., 2017), using methods consistent with those reported previously (Kucyi et al., 2021). We used both leave-one-trial-out and five-fold cross-validation to verify the stability of results. To generate features for predictive modeling, we extracted BOLD preprocessed time series from 300 regions in the Schaefer atlas from each fMRI experience-sampling run. We then correlated the time series between all region-pairs and Fisher z-transformed the Pearson correlation coefficients to generate a functional connectivity matrix for each trial within the 30-s window (28 frames) preceding the appearance of each thought probe (Kucyi et al., 2021). For each cross-validation fold, we identified edges (pairs of regions) with a correlation, positive or negative, to mind-wandering ratings that surpassed an uncorrected threshold of *p* < 0.01 (two-tailed). This step generated a “positive mask” and “negative mask,” each composed of edges identified as features associated with mind-wandering. For each trial, we computed the dot product between the functional connectivity matrix and each mask. We then summed all positive and negative mask edge values. We calculated single network strength value based on the difference between summed negative mask and summed positive mask edge values. Using all trials within a given fold, we fit a linear model with network strength as the independent variable and mind-wandering as the dependent variable. This model was then applied to predict mind-wandering for the held-out trial(s), and predicted ratings were correlated with observed mind-wandering ratings. For five-fold cross-validation, the procedures described above were repeated 120 times with randomized cross-validation fold schemes, and the mean correlation between predictive versus observed mind-wandering was computed across iterations. To determine statistical significance of CPM, we performed 1,000 permutation tests to generate null correlation values for predicted versus observed mind-wandering. Significance was set at *p*_*perm*_ < 0.05.

In control analyses, we repeated the CPM procedures, but during model training, we performed partial correlations between predictive versus observed mind-wandering, controlling for the following variables: (a) time on task within run; (b) trial number within session; and (c) head motion (i.e., mean framewise displacement within 30-s window prior to thought probe). For null distribution computation (based on 1,000 permutations), mind-wandering ratings and control variable values were jointly shuffled.

Recent work has suggested that alternative predictive modeling methods may outperform CPM in producing reliable functional connectivity patterns (Sripada et al., 2019; Taxali et al., 2021). To ensure that our results were not limited by the CPM approach, we performed brain basis set modeling (Kessler et al., 2016), which reduces input features (i.e., edge) using principal components analysis before conducting linear regression of the outcome scores. We chose to reduce the data to 75 components as per previous studies (Sripada et al., 2019; Taxali et al., 2021). Significance was assessed with permutation tests as done in the CPM analyses.

### Analyses of CPM Feature Importance

To interpret each individualized mind-wandering CPM, we generated positive and negative CPM masks within each subject using a single fold that included all trials (i.e., we correlated mind-wandering rating versus functional connectivity for each edge and set an uncorrected threshold of *p* < 0.01, two-tailed, for positive and negative masks). We mapped the positive and negative CPM mask edges on to the Schaefer 300 atlas, with each node assigned to one of seven standard Yeo-Krienen networks (Yeo et al., 2011). We quantified the number of edges belonging to each intra- or inter- network pair (28 total network pairs).

Next, we performed statistical tests of feature importance using CPM with computational network lesions. We performed these analyses with the following two variations: (a) Single-network lesion: All edges assigned to one Yeo-Krienen network were removed during CPM training and testing. (b) Single-network retention: All edges that were not assigned to a specific Yeo-Krienen network were removed during CPM training and testing. Computationally lesioning all but one of the seven Yeo-Krienen networks, we generated individualized subject CPMs based on each of the isolated seven networks to determine which, if any, network, could predict mind-wandering independently. Statistical significance was assessed in the same manner as in the main CPM analyses with 1,000 permutation tests and significance set at *p*_*perm*_ < 0.05.

To further illustrate individual variability in features contributing to CPMs, we plotted node degree using the BioImage Suite Connectivity Visualization Tool (https://bioimagesuiteweb.github.io/webapp/connviewer.html). These plots were based on the main within-subject CPM analyses performed using the Shen atlas of 268 regions (Shen et al., 2013) rather than the Schaefer 300 atlas (CPM performance was very similar regardless of atlas choice; see Supporting Information Table S2A). The node degree plots illustrate, for each node in the atlas, the total number of edges that the node participated in for positive and negative CPM masks (Figure 5D).

### Cross-Subject CPM

We tested model generalizability across subjects using cross-subject testing of the personalized models generated by idiographic CPM. To obtain within-subject CPMs that could be tested out of sample in other subjects, we generated a CPM linear model within each subject using a single fold that included all trials. We applied the linear model from each individualized CPM to generate predicted mind-wandering ratings for the remaining two subjects (i.e., if using the CPM trained on Subject 1, the linear model was applied to Subjects 2 and 3, respectively). In external data from test subjects, we computed network strength based on the dot products of trial-wise functional connectivity matrices and the positive and negative edge masks from the model training subject. Network strength was computed based on subtraction of negative from positive edge scores. We computed predicted mind-wandering scores based on linear parameters obtained in the model training subject. We then calculated the Spearman’s rank correlation coefficient for the predicted versus observed mind-wandering ratings (significance set at *p* < 0.05, two-tailed).

### Testing Population-Derived CPMs

We tested the ability of two previously published, population-derived CPMs (i.e., those that combined features derived from multiple individuals during model training) to predict mind-wandering within each of our densely sampled subjects. These two CPMs were (a) the stimulus-independent, task-unrelated thought CPM (SITUT-CPM) (Kucyi et al., 2021), publicly available at https://github.com/swglab/CPM_CONN; and (b) the sustained attention CPM (SA-CPM) (Rosenberg et al., 2016), publicly available at https://github.com/monicadrosenberg/Rosenberg_PNAS2020/tree/master. We tested the SITUT-CPM because this model was predictive (at a group level) of within-subject mind-wandering away from a continuous performance task, within both healthy adults and adults diagnosed with attention-deficit/hyperactivity disorder (Kucyi et al., 2021). The SA-CPM was shown to be predictive of sustained attention ability, both across (Rosenberg et al., 2016) and within (Rosenberg et al., 2020) individuals, and showed a (weak) negative correlation with SITUT-CPM expression (Kucyi et al., 2021).

To test the population-derived CPMs, we computed trial-wise network strength based on the dot products of trial-wise functional connectivity matrices and the positive and negative edge masks from the respective CPM. Trial-wise network strength was computed based on subtraction of negative from positive edge scores. We then computed the Spearman’s rank correlation coefficient between CPM network strength and observed mind-wandering ratings (significance set at *p* < 0.05, two-tailed). To test the SITUT-CPM, features were derived from the Schaefer 300-region atlas (as used in the idiographic CPM analyses). The SA-CPM was created based on the Shen atlas of 268 regions (Shen et al., 2013), and so the same atlas was used to extract features when testing this model.

## SUPPORTING INFORMATION

Supporting information for this article is available at https://doi.org/10.1162/netn_a_00387.

## AUTHOR CONTRIBUTIONS

Aaron Kucyi: Conceptualization; Formal analysis; Methodology; Software; Supervision; Visualization; Writing – original draft; Writing – review & editing. Nathan Anderson: Formal analysis; Software; Visualization; Writing – review & editing. Tiara Bounyarith: Formal analysis; Visualization; Writing – review & editing. David Braun: Formal analysis; Visualization; Writing – review & editing. Lotus Shareef-Trudeau: Writing – original draft; Writing – review & editing. Isaac Treves: Formal analysis; Visualization; Writing – review & editing. Rodrigo M. Braga: Software; Supervision; Writing – review & editing. Po-Jang Hsieh: Conceptualization; Funding acquisition; Investigation; Methodology; Project administration; Supervision; Writing – review & editing. Shao-Min Hung: Conceptualization; Data curation; Formal analysis; Investigation; Methodology; Project administration; Resources; Writing – review & editing.

## FUNDING INFORMATION

Aaron Kucyi, National Institute of Mental Health (https://dx.doi.org/10.13039/100000025), Award ID: R21MH129630. Nathan Anderson, National Institute of Mental Health (https://dx.doi.org/10.13039/100000025), Award ID: T32NS047987.

## Supplementary Material



## TECHNICAL TERMS

Group-to-individual generalizability: How well aggregated group data reflect the individuals who comprise it.
Precision functional mapping: A brain imaging approach that uses individual-level data to characterize personalized functional brain organization in detail.
Dense sampling: Data collection method of obtaining a larger quantity of data points per individual, allowing for more precise individual-level analysis.
Functional localizer: Refers to an independent task that localizes brain regions of interest to guide interpretation of results in the main experiment.
Hierarchical Bayesian modeling (HBM): A statistical model for analyzing data structures where observations are nested within groups. Submodels form the hierarchy, integrated through Bayes’s theorem.
Functional network: A set of brain regions that are mutually engaged to perform some function.
General linear model (GLM): A statistical model for analyzing the extent that multiple variables contribute to a continuous variable of interest through various linear regressions.
Connectome-based predictive modeling (CPM): A supervised machine learning method for mapping brain-behavior relationships from whole-brain functional connectivity.
Edge: Describes pairwise interactions between regions (nodes) in brain networks.
Biomarker: Features within an individual indicating the presence or absence of a disease state; can inform diagnosis, prognosis, and treatment.
