# Supplementary material for: Individual variability in neural representations of mind-wandering
Source: Netw Neurosci. 2024 Oct 1;8(3):808–36. doi: 10.1162/netn_a_00387 (PMC11349032; doi:10.1162/netn_a_00387)
Supplement: Supplementary file 1 [file netn-8-3-808-s001.pdf]

Supplementary Information

Individual variability in neural representations of mind wandering

Kucyi et al.

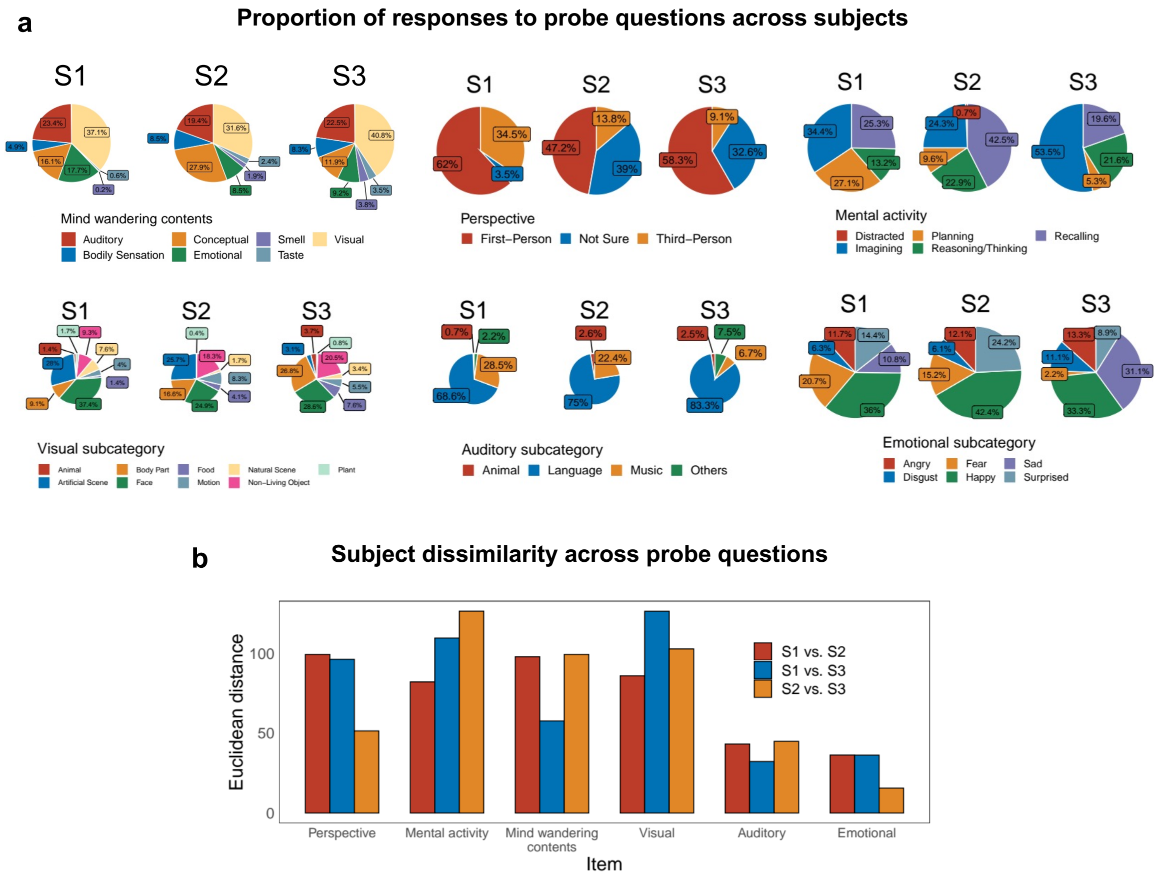

Figure S1. Individual differences in responses to mind wandering subcategory probes. a) For each subject, proportion of responses to each option for six probe questions concerning contents, perspective, type of mental activity, visual subcategory, auditory subcategory, and emotional content. **b**) Euclidean distance between response counts for each pair of subjects and each probe question. The distance between two subjects for a probe question was computed by first tallying the number of responses to each option for each subject, and then computing the Euclidean distance between the two tallies.

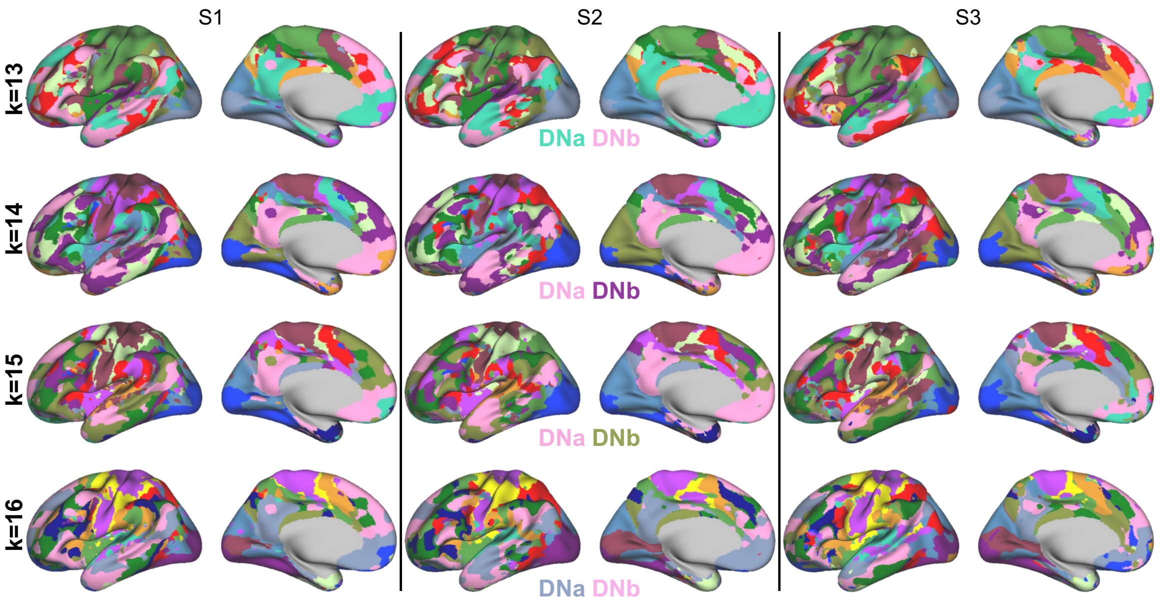

Figure S2. Personalized functional networks estimated with multi-session hierarchical Bayesian modeling (MS-HBM) with multiple clustering solutions. For each subject and clustering solutions set between 13 and 16 (see Fig. 3 for 17 cluster solution), cortical networks obtained with MS-HBM are shown. Consistency was found across solutions in identifying two subnetworks of the default mode network, default network A (DNa) and B (DNb).

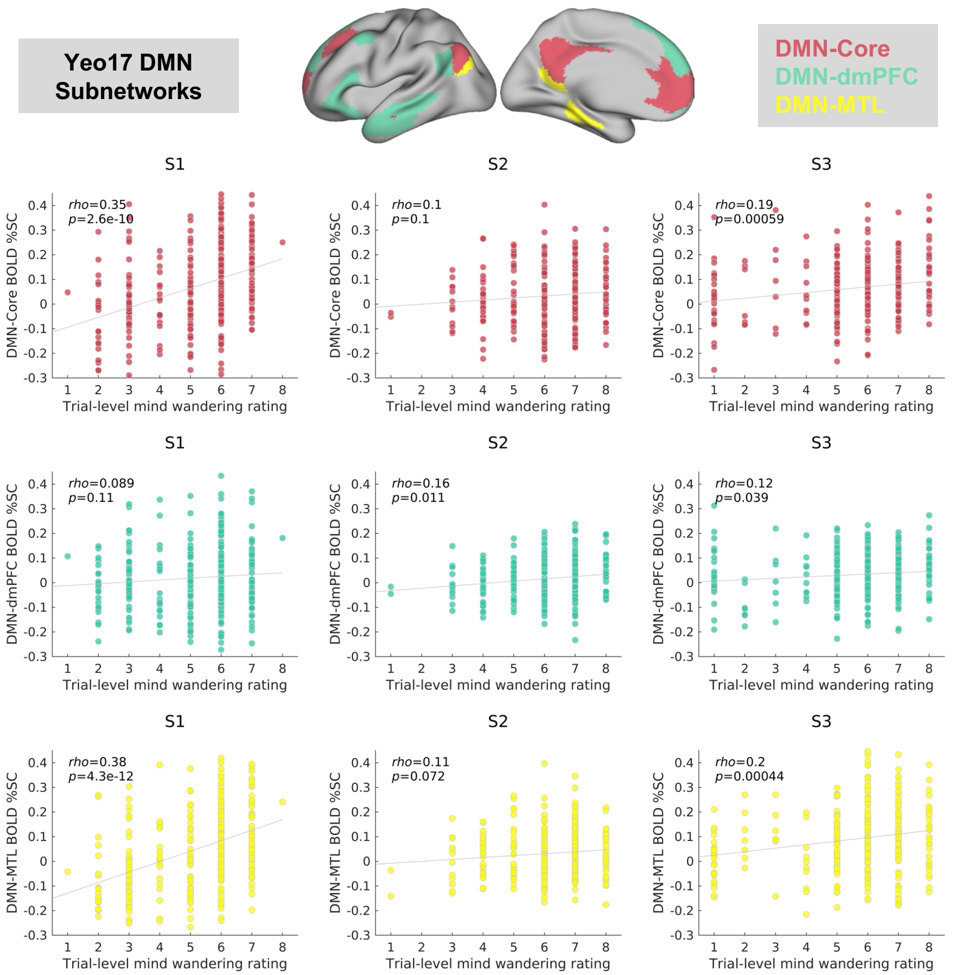

Figure S3. Correlations between mind wandering and activation within standard-space subnetworks of the default mode network (DMN). The top cortical surface plots illustrate the locations of standard-space subnetworks from the population-derived Yeo-Krienen 17-network atlas. These networks include the DMN-core, DMN-dorsomedial prefrontal cortex (dmPFC) and DMN-medial temporal lobe (MTL) subsystems. Scatter plots show correlations within each subject between trial-by-trial mind wandering and the median of blood oxygenation level dependent (BOLD) percent signal change (%SC) within 10-second pre-thought probe periods for a given DMN subnetwork. The top, middle and bottom rows of plots, respectively, show results for the DMN-Core, DMN-dmPFC, and DMN-MTL subnetworks.

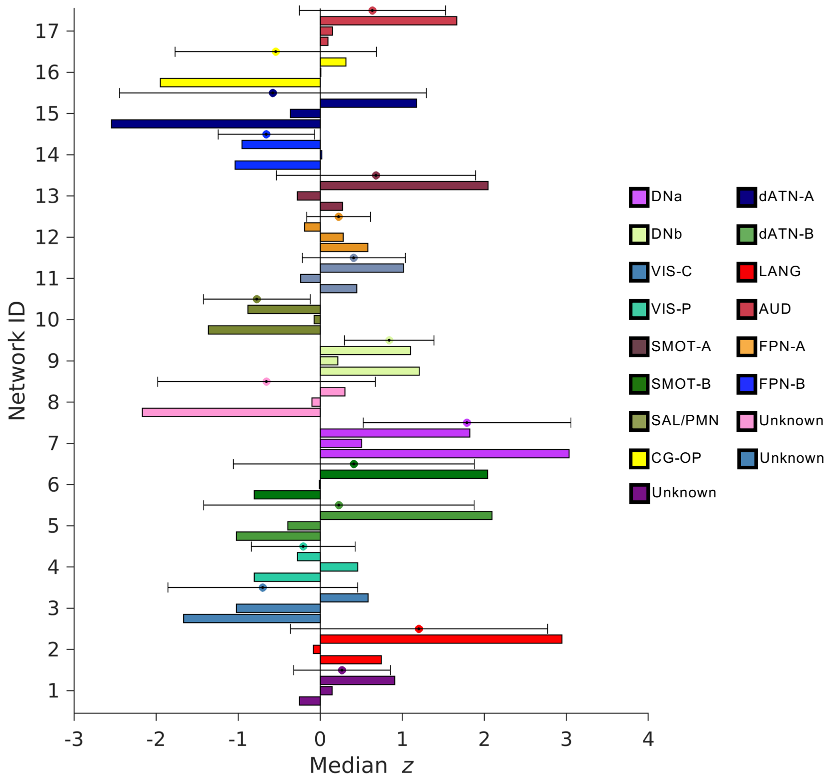

Figure S4. Brain activation associated with mind-wandering within 17 networks, shown for all three subjects in a single plot. For each network (derived in each subject with precision functional mapping) shown in a unique color, the median z score is plotted as obtained from a general linear model (GLM) analysis in which mind-wandering rating was a regressor in modeling 10-second periods prior to thought probes. Bars are shown for single subjects, dots indicate mean across subjects, and error bars indicate standard deviation across subjects. AUD = auditory; CG-OP = cigulo-opercular; dATN = dorsal attention network; DN = default network; FPN = frontoparietal network; LANG = language; SAL/PMN = salience/parietal memory network; SMOT = somatomotor; VIS-C = visual central; VIS-P = visual peripheral.

Table S1. **Control analyses accounting for head motion and time on task. a)** Correlations between trial-wise mind wandering and head motion (i.e., mean framewise displacement in 30-second windows prior to thought probes), time within run (i.e., onset time of thought probe from start of each run), and trial order within session for all subjects. **b)** Partial correlations between trial-wise mind wandering and DNa activation (BOLD %SC in 10-second windows prior to thought probes) controlling for motion and time on task. **c)** Same as b) but for DNb instead of DNa. **d)** Results of within-subject connectome-based predictive modeling of mind wandering (five-fold cross validation), using partial correlations within each cross-validation fold to account for head motion and time-on-task. Mean *r* values indicate the average of predicted versus observed mind wandering correlations across 120 cross-validation iterations, and *P* values are based on 1000 permutations. **P*<0.05. DNa = default network A; DNb = default network B.

a) Correlation with mind wandering ratings for:

	**Motion (framewise displacement)**		**Time within run**		**Trial order within session**	
	Spearman's ρ	*P* value	Spearman's ρ	*P* value	Spearman's ρ	*P* value
**S1**	0.18*	0.0011	-0.064	0.26	0.057	0.31
**S2**	0.096	0.12	-0.035	0.58	-0.18*	0.004
**S3**	0.00016	0.98	0.097	0.084	-0.018	0.74

b) Partial correlation between DNa activation and mind wandering, controlling for:

	**Motion (framewise displacement)**		**Time within run**		**Trial order within session**	
	Spearman's ρ	*P* value	Spearman's ρ	*P* value	Spearman's ρ	*P* value
**S1**	0.43*	6.6x10^{-16}	0.44*	4.4x10^{-16}	0.43*	6.7x10^{-16}
**S2**	0.11	0.088	0.11	0.078	0.12	0.063
**S3**	0.33*	2.7x10^{-9}	0.32*	5.2x10^{-9}	0.33*	2.6x10^{-9}

c) Partial correlation between DNb activation and mind wandering, controlling for:

	**Motion (framewise displacement)**		**Time within run**		**Trial order within session**	
	Spearman's ρ	*P* value	Spearman's ρ	*P* value	Spearman's ρ	*P* value
**S1**	0.13*	0.024	0.13*	0.024	0.12*	0.030
**S2**	0.11	0.071	0.12	0.057	0.13*	0.034
**S3**	0.28*	4.6x10^{-7}	0.28*	5.5x10^{-7}	0.28*	4.9x10^{-7}

d) Predicted vs. observed mind wandering for within-subject connectome-based predictive modeling (five-fold cross validation), controlling for:

	**Motion (framewise displacement)**		**Time within run**		**Trial order within session**	
	Mean *r*	P_{perm}	Mean *r*	P_{perm}	Mean *r*	P_{perm}
**S1**	0.31*	0.001	0.32*	0.001	0.32*	0.001
**S2**	0.16*	0.021	0.16*	0.030	0.16*	0.018
**S3**	0.38*	0.001	0.38*	0.001	0.38*	0.001

Table S2. **Alternative predictive modeling results. a)** Results of within-subject connectome-based predictive modeling of mind wandering (five-fold cross validation), using the Schaefer 300-region and Shen 268-region atlases. **b)** Results of within-subject brain basis set (BBS) modeling of mind wandering (five-fold cross validation), using the Schaefer 300-region and Shen 268-region atlases. *P<0.05

a) CPM

	**Schaefer300**		**Shen268**	
	Mean r	P_{perm}	Mean r	P_{perm}
**S1**	0.31*	0.001	0.31*	0.001
**S2**	0.16*	0.025	0.20*	0.008
**S3**	0.38*	0.001	0.39*	0.001

b) BBS

	**Schaefer300**		**Shen268**	
	Mean r	P_{perm}	Mean r	P_{perm}
**S1**	0.38*	0.001	0.27*	0.001
**S2**	0.11	0.074	0.22*	0.007
**S3**	0.29*	0.001	0.34*	0.001
